# Event-Based Color Segmentation With a High Dynamic Range Sensor

**DOI:** 10.3389/fnins.2018.00135

**Published:** 2018-04-11

**Authors:** Alexandre Marcireau, Sio-Hoi Ieng, Camille Simon-Chane, Ryad B. Benosman

**Affiliations:** Institut National de la Santé et de la Recherche Médicale, UMRI S 968, Sorbonne Universites, UPMC Univ Paris 06, UMR S 968, Centre National de la Recherche Scientifique, UMR 7210, Institut de la Vision, Paris, France

**Keywords:** event-based signal processing, AER, color segmentation, tracking, silicon retina

## Abstract

This paper introduces a color asynchronous neuromorphic event-based camera and a methodology to process color output from the device to perform color segmentation and tracking at the native temporal resolution of the sensor (down to one microsecond). Our color vision sensor prototype is a combination of three Asynchronous Time-based Image Sensors, sensitive to absolute color information. We devise a color processing algorithm leveraging this information. It is designed to be computationally cheap, thus showing how low level processing benefits from asynchronous acquisition and high temporal resolution data. The resulting color segmentation and tracking performance is assessed both with an indoor controlled scene and two outdoor uncontrolled scenes. The tracking's mean error to the ground truth for the objects of the outdoor scenes ranges from two to twenty pixels.

## 1. Introduction

Primates' ability to discriminate colors is advantageous for survival (Dominy and Lucas, 2001). They use it for long-range detection of edible food in forest environments. This ability is nowadays exploited by humans to efficiently communicate information: road signs, merchandizing and maps are a few examples. Consequently, machine vision systems meant to interface with humans or mimic their vision often rely on colors (Trémeau et al., 2008). Applications include traffic sign recognition (Bahlmann et al., 2005), skin detection (Kakumanu et al., 2007), visual saliency modeling (van de Weijer et al., 2006) or vehicle color classification (Hsieh et al., 2015). However, detecting colored objects in a scene remains a challenge for such systems: even though a considerable amount of research has been carried out on color segmentation, *ad hoc* techniques are still required to solve many problems. The large amount of recently published works (Vantaram and Saber, 2012) tends to demonstrate that automated color segmentation is far from being solved. Current state-of-the-art methods for color video segmentation rely on a model describing tracked objects: superpixels (Pun and Huang, 2016), graphs (Rother et al., 2004; Grundmann et al., 2010; Lezama et al., 2011) or local classifiers (Bai et al., 2010; Lee et al., 2011). These methods yield robust and accurate results, but require heavy computations. Other methods rely on clustering techniques, especially *Mean Shift* derivatives, to segment colors (Fukunaga and Hostetler, 1975; Cheng, 1995). The results quality and very high computational costs drove research on speed optimization and complexity reduction through structuring of the feature space (Guo et al., 2006; Paris and Durand, 2007; Xiao and Liu, 2010), dynamic bandwidth selection (Yang et al., 2003) or kernel choice improvements (Comaniciu, 2003). Despite these optimizations, state-of-the-art methods still require large computations preventing their use for real-time or low-power applications. This need derives from the dense and exceedingly redundant visual information provided by conventional, frame-based cameras. By contrast, the human eye contains several neuron layers known to reduce redundancy and participate in color information processing (Johansson, 2004).

This work introduces a new direction for color segmentation, with algorithms operating on high temporal resolution data (down to one microsecond) provided by neuromorphic event-based cameras which mimic the human eye. These sensors are based on pixels operating independently. Instead of capturing information at a fixed frame-rate, with no relation to the visual information source (Lichtsteiner et al., 2006), each pixel optimizes its sampling depending on the visual information it receives. If the scene changes quickly, the pixel samples information with a high adaptive rate. Otherwise, the pixel stops acquiring redundant data and goes idle until luminance changes in its field of view, therefore contributing to information processing. The sensor does not require a common frame clock, since event-based cameras' pixels are independent and autonomous. A variety of such sensors were developed in the past few years: temporal contrast vision sensors detecting relative luminance changes (Lichtsteiner et al., 2006; Posch et al., 2008, 2011), gradient-based sensors detecting static edges (Delbruck, 1993), edge-orientation sensitive devices (Etienne-Cummings et al., 1997) and optical flow sensors (Krammer and Koch, 1997).

**Figure 1:**
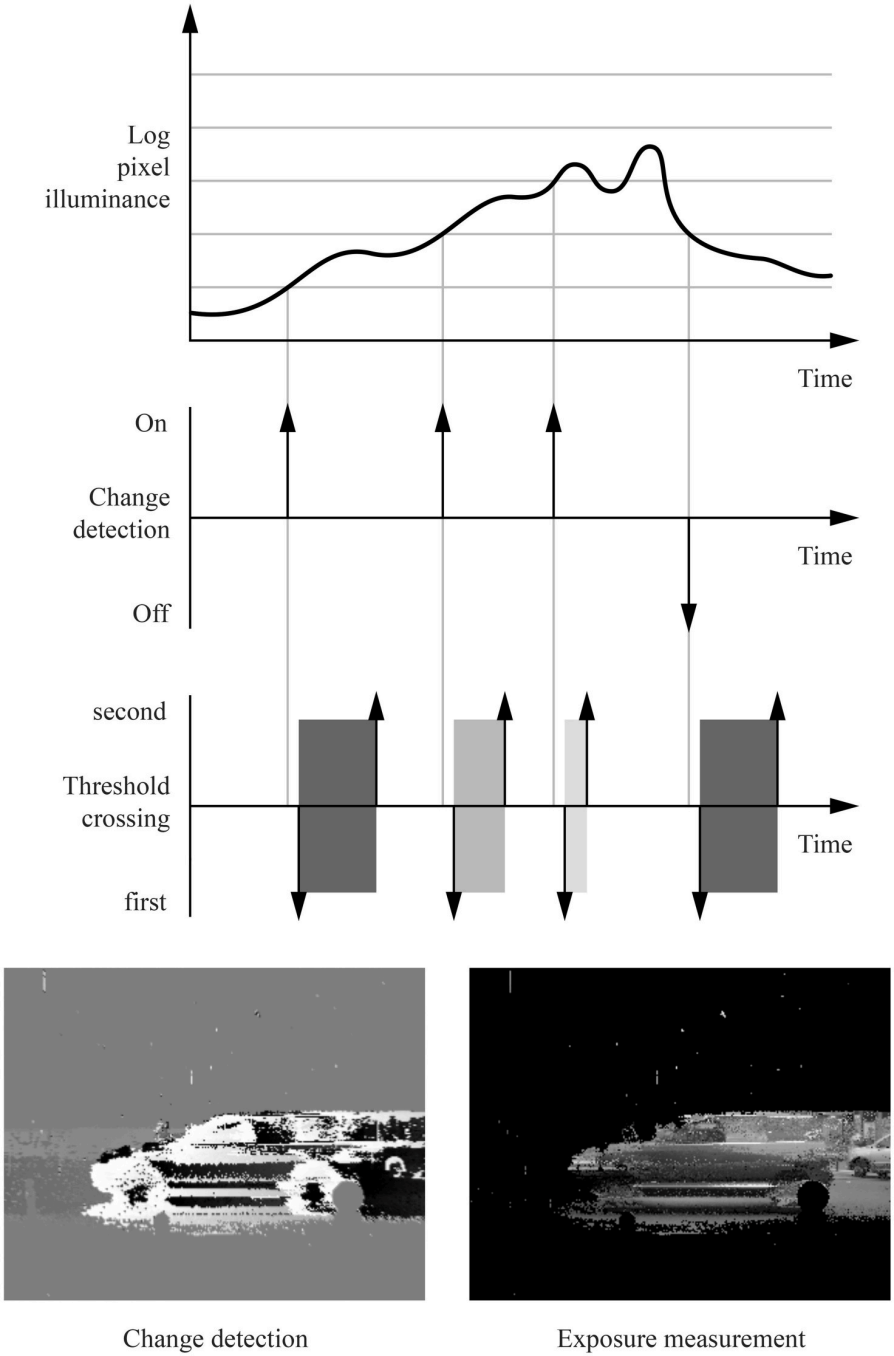
The ATIS is an asynchronous, event-based camera with independent pixels. This figures illustrates the behavior of a single pixel. When the logarithm of the luminance captured by the pixel crosses a threshold, light integration for the pixel starts. The exposure measurement's duration is proportional to the inverse of the luminance, and is notified by two events called threshold crossings: the first one is sent when the integration begins, the second one when it ends.

The *Asynchronous Time-based Image Sensor* (Posch et al., 2011), or ATIS, used in this paper is an asynchronous camera that contains an array of autonomously operating pixels that combine an asynchronous change detection circuit and a separate exposure measurement circuit, the latter being triggered by the former. Each pixel independently and continuously monitors its field of view. The detection of luminance change triggers a local light integration, as illustrated Figure 1. The information is output asynchronously with the pixel coordinates, hence providing the new gray level. Consequently, the scene is not acquired frame-wise, but rather continuously and locally, conditionally on visual information changes. In other words, only information that is relevant—because unknown—is acquired, transmitted, stored and eventually processed by machine vision algorithms. Pixel acquisition and readout times range from milliseconds to microseconds, resulting in temporal resolutions equivalent to conventional sensors running at tens to hundreds of thousands frames per second. The sparse nature of the generated visual data benefits subsequent processing in terms of speed and power consumption. Moreover, the data's high temporal resolution allows for simplifying assumptions, with complex behaviors emerging from simple, high-speed algorithms. The event-based formulation of vision problems in the time domain has already produced striking results for many computer vision algorithms, such as stereo-vision (Rogister et al., 2012; Carneiro et al., 2013), optical flow (Benosman et al., 2014) or tracking (Ni et al., 2012).

**Figure 2:**
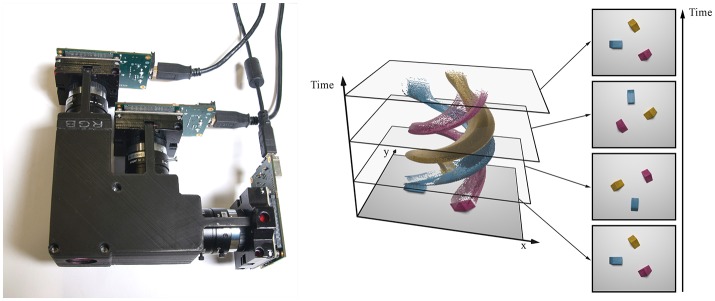
The three-chip event-based camera is an assembly of three ATIS cameras **(Left)**. The cameras share the same field of view. Reconstructed color events can be visualized as a spatio-temporal point-cloud **(Right)**. There are as many color points in the four frames (far right) as in the whole point cloud.

The benefits of event-based cameras make them well-suited candidates to overcome existing limitations in automated color segmentation. To our knowledge, only two attempts at building a color event-based sensor have been made. The pixel proposed by Berner and Delbruck (2011) is sensitive to both luminance and wavelength changes. Moeys et al. (2017) added a Bayer matrix to an existing DVS sensor. However, both sensors are only sensitive to relative luminance changes, and were not illustrated with concrete applications. Using the ATIS capacity to acquire absolute luminance in an event-based manner, we present in this paper both a functional event-based color sensor, illustrated Figure 2, and its application for segmenting colored objects with simple processing techniques requiring little computation power. The absolute luminance information allows for a robust and computationally cheap color segmentation based on clustering, unlike change detectors which need to rely on edges detection. We evaluate the sensor's ability to track colored shapes, using a real-time on-line algorithm. Thanks to the nature of the data generated by event-based cameras, tracking can be implemented with a moving mean algorithm (Drazen et al., 2011), which requires very little computational power. More complex and robust methods have been devised (Lagorce et al., 2015; Reverter Valeiras et al., 2015) for more demanding applications.

## 2. Materials and methods

### 2.1. Three-chip event-based camera

**Figure 3:**
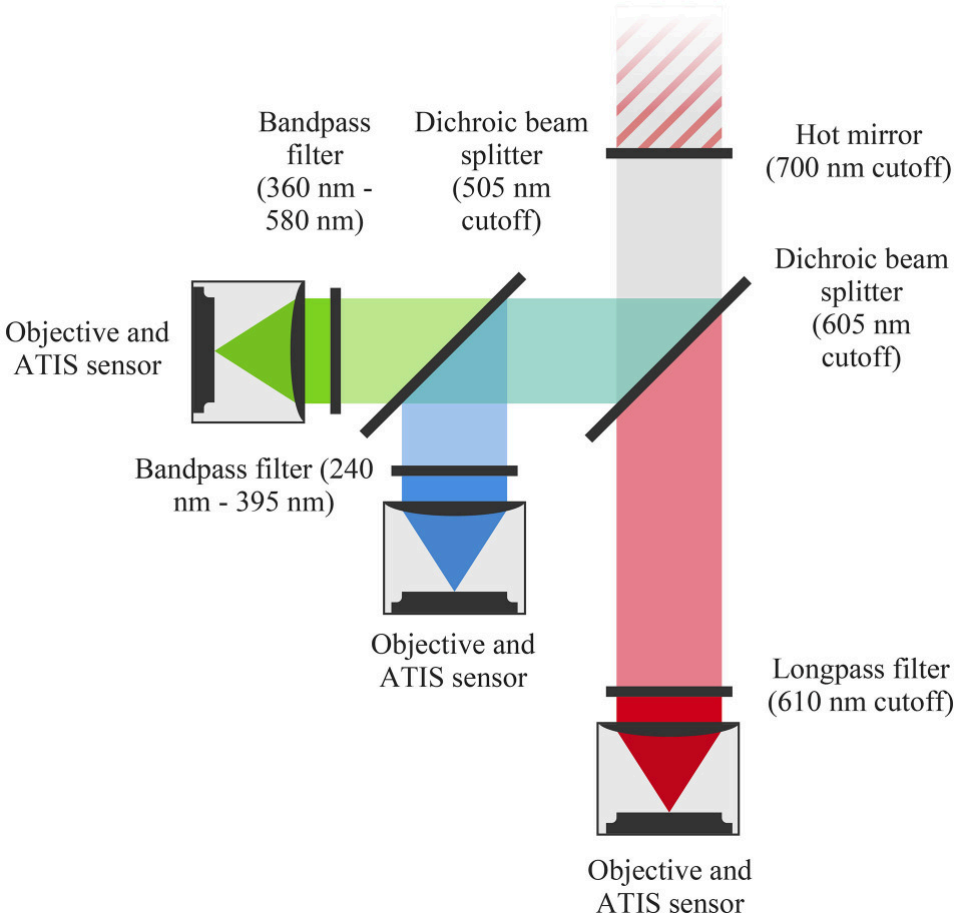
Our three-chip event-based camera uses dichroic filters to split the light beam and ATIS cameras to record the scene as events. We use an objective for each camera instead of a single one in order to reduce the flange distance (constrained by the sensors' size).

We build an event-based color sensor as an association of three ATIS cameras acquiring red, green and blue light exposures. The sensor captures light through a hot mirror reflecting infra-red light. A beam splitter directs photons with wavelengths larger than 605 nm toward the red sensor. The other photons are reflected toward a second beam splitter, which directs photons with wavelengths smaller than 505 nm toward the blue sensor. The remaining photons are directed toward the green sensor. Before hitting the red, green and blue sensors, photons cross band-pass filters which mimic the filtering functions of conventional Bayer matrices' pixels. Each sensor uses a C-mount objective, as the sensors dimensions prevent using a common objective placed behind the hot-mirror. Figure 3 illustrates the assembly.

In order to account for the mechanical imperfections of the prototype, a spatial calibration step is required to make sure that the color sensor's cameras share the same field of view. We capture a checker board with the sensor before each recording, and compute the homography linking the green and blue cameras to the red one. The homography is computed by determining the direct linear transformation on normalized points (Hartley, 1995), which were extracted from a reconstructed image of the checker board using corner detection and structure recovery (Geiger et al., 2012). This spatial calibration is valid only for objects within the checker board's plan. However, we observe a good pixel matching for objects in other plans as well, as the fields of view differences are small compared to the pixels size.

### 2.2. Color events

After applying the spatial calibration step, the red sensor's pixel with index *i* has the same field of view as the green and blue sensors' pixels with index *i*. We call *pixel with index i of the color sensor* the virtual pixel combining the red, green and blue pixels with index *i*. The signal captured by this pixel can be modeled as a continuous ℝ^3^ function *s*_*i*_ of the time *t*:

(1) $$\begin{array}{r} \begin{array}{cc} s_{i}:\mathbb{R} & \to {\mathbb{R}}^{3}\\ t & \mapsto \left(r,g,b\right) \end{array} \end{array}$$

where *r*, *g*, and *b* are the red, green and blue components intensities of the signal. *i*, the pixel's index, is in the range [1, *n*], where *n* is the sensor's number of pixels. We want the color sensor to generate events *e*_*i,t*_ defined by the tuple of attributes:

(2) $$\begin{array}{r} e_{i,t}=\left(i,t,r,g,b\right) \end{array}$$

Assuming an initial value *s*_*i*_(*t*_0_) = (*r*_0_, *g*_0_, *b*_0_), the pixel with index *i*'s first event should be generated at the time *t*_1_ such that *s*_*i*_(*t*_1_) = (*r*_1_, *g*_1_, *b*_1_) and the distance in ℝ^3^ between (*r*_0_, *g*_0_, *b*_0_) and (*r*_1_, *g*_1_, *b*_1_) is larger than a tunable threshold. The distance function should not be the euclidean distance in order to mimic human perception, which is highly non-linear in RGB space (Cheng et al., 2001).

**Figure 4:**
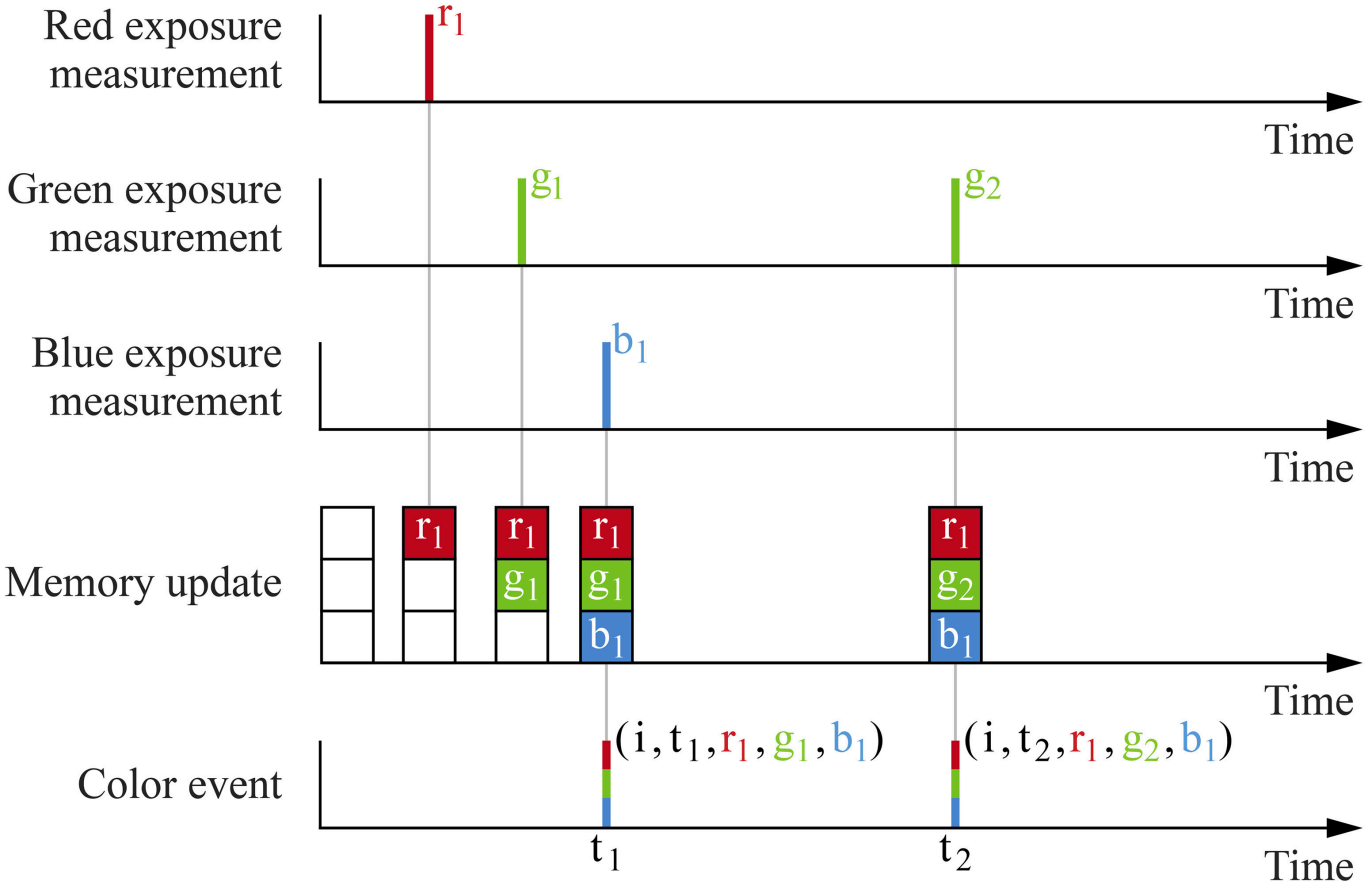
Events generated by the color sensor's three cameras' pixel *i* are merged to generate color events. Every time an exposure measurement is generated by one of the ATIS cameras, it is stored in memory. Then, a color event is dispatched using the current memory value.

The ATIS-based three-chip camera's pixels do not yield the *s*_*i*_ signal directly. Instead, the camera associated with each color component generates an independent stream of events. Since ATIS cameras yield exposure measurements with a delay inversely proportional to the measured exposure, it is not possible to detect temporally coinciding events to generate color events. Therefore, we associate each color sensor's virtual pixel with a memory space storing the three color components. Every time an event is generated by one of the color component cameras, the memory is updated and a color event based on the current memory value is dispatched. This mechanism is illustrated Figure 4.

### 2.3. Color model

We consider color object tracking as a first practical application of the event-based color sensor. Given a pre-determined set of uniformly colored objects, we want to determine the position of each object in an scene at every moment. We use a two-step approach : first, we build a statistical color signature for each object using a labeled scene. Then, events from an unknown scene are matched against the statistical models and associated with the closest signature.

In order to reduce the required amount of computation for each event, we reduce the problem's dimensionality by converting color events from the RGB space to the CIEL^*^a^*^b^*^ space. We use only the a^*^ and b^*^ components of the latter. For each object, we gather events from the labeled scene and project them to the a^*^b^*^ plane of the CIEL^*^a^*^b^*^ space. We use a bivariate normal distribution as a statistical model for describing these points.

**Figure 5:**
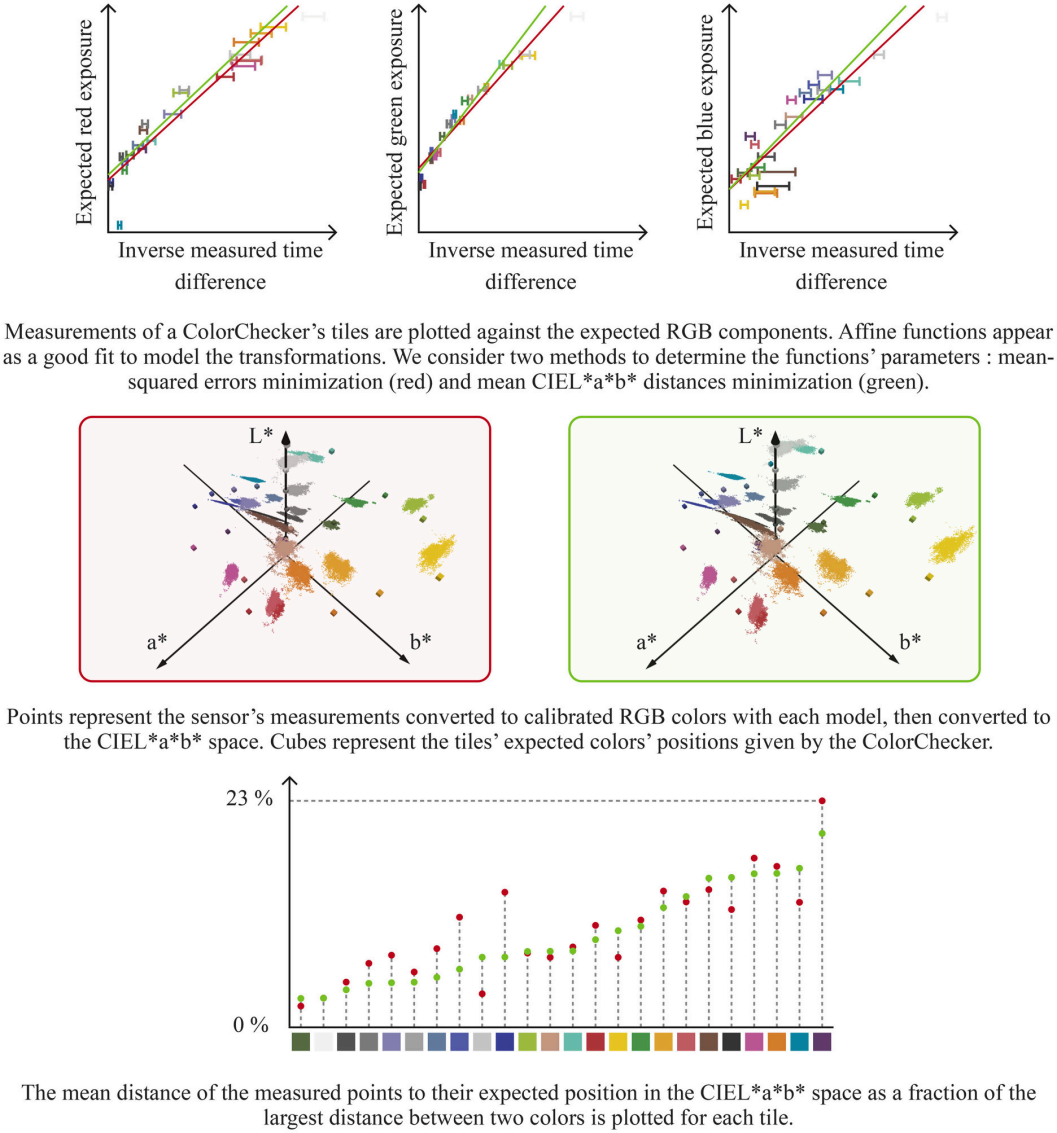
Converting the sensor's color events to the CIEL*a*b* space requires a transformation model. We use a Macbeth ColorChecker to compare the expected color values with the measured time differences. We consider two strategies for finding our model's parameters : mean squared error (red) and non-linear optimization to minimize the distance between expected and measured colors in the CIEL*a*b* space (green). The latter yields a better fit for blue and purple colors in the a*b* plane. On the a*b* plane figures, squares represent the expected colors and circles represent the mean measured value. The represented Macbeth ColorCheckers were captured by our sensor.

Converting events from the RGB space to the CIEL^*^a^*^b^*^ space requires a color calibration step. ATIS cameras send exposures as a pair of threshold-crossing events to the computer. The actual exposure is—as a first approximation—proportional to the inverse of the time difference between the two thresholds-crossing events. We use a Macbeth ColorChecker to evaluate the required proportionality factor between the time difference inverse and red, green and blue components in RGB space. We observe that the expected red, green and blue values given by the Macbeth ColorChecker as functions of the measured inverse threshold-crossing time differences are well described by affine functions, as shown Figure 5. The need for affine functions instead of linear functions can be attributed to the sensor imperfections, including the pixels' dark current. We calculate the affine regression by minimizing the mean squared error for each color component. This method yields good results for displaying the sensor's measurements using an RGB screen. Estimated red, green and blue components can be used to determine the CIEL^*^a^*^b^*^ color components using several non-linear relations (Jain, 1989).

However, when using this model to convert the measured colors to the CIEL^*^a^*^b^*^ space, we observe a poor fit with the values given by the Macbeth ColorChecker. The difference can be attributed to the uncorrelated regression applied to each component and the ATIS cameras' noise. Therefore, we use the Nelder-Mead simplex algorithm (Lagarias et al., 1998) to optimize the six parameters of the three color components' affine regressions. We minimize the distances between the expected colors given by the Macbeth ColorChecker and the measured points in CIEL^*^a^*^b^*^ space. Since the CIEL^*^a^*^b^*^ space is perceptually uniform, this method yields the best compromise for converting the measured Macbeth ColorChecker's colors to the CIEL^*^a^*^b^*^ space with regards to human perception. Figure 5 shows the two methods results.

### 2.4. Signatures

We consider the sequence *S* of *n* color events associated with a uniformly colored object:

(3) $$\begin{array}{r} S=\left(\left(i_{k},t_{k},r_{k},g_{k},b_{k}\right),k\in ⟦0,n-1⟧\right) \end{array}$$

We define *S*_*ab*_ as the sequence of pairs (*a, b*) obtained by converting each color event from the sequence *S* to the CIEL^*^a^*^b^*^ space:

(4) $$\begin{array}{r} S_{ab}=\left(\left(a_{k},b_{k}\right),k\in ⟦0,n-1⟧\right) \end{array}$$

We call *signature* of the considered object the bivariate normal distribution N(**μ**, **Σ**) estimated from the *S*_*ab*_ sequence:

(5) $$\begin{array}{l} \mu =\left(\begin{array}{c} \mu_{a}\\ \mu_{b} \end{array}\right)=\frac{1}{n}\sum_{k\;=\;0}^{n-1}\left(\begin{array}{c} a_{k}\\ b_{k} \end{array}\right)\\ \Sigma =\left(\begin{array}{cc} \sigma_{a}^{2} & \sigma_{ab}\\ \sigma_{ab} & \sigma_{b}^{2} \end{array}\right)\\ \;=\frac{1}{n-1}\sum_{k\;=\;0}^{n-1}\left(\left(\begin{array}{c} a_{k}\\ b_{k} \end{array}\right)-\mu \right){\left(\left(\begin{array}{c} a_{k}\\ b_{k} \end{array}\right)-\mu \right)}^{\text{T}} \end{array}$$

**Figure 6:**
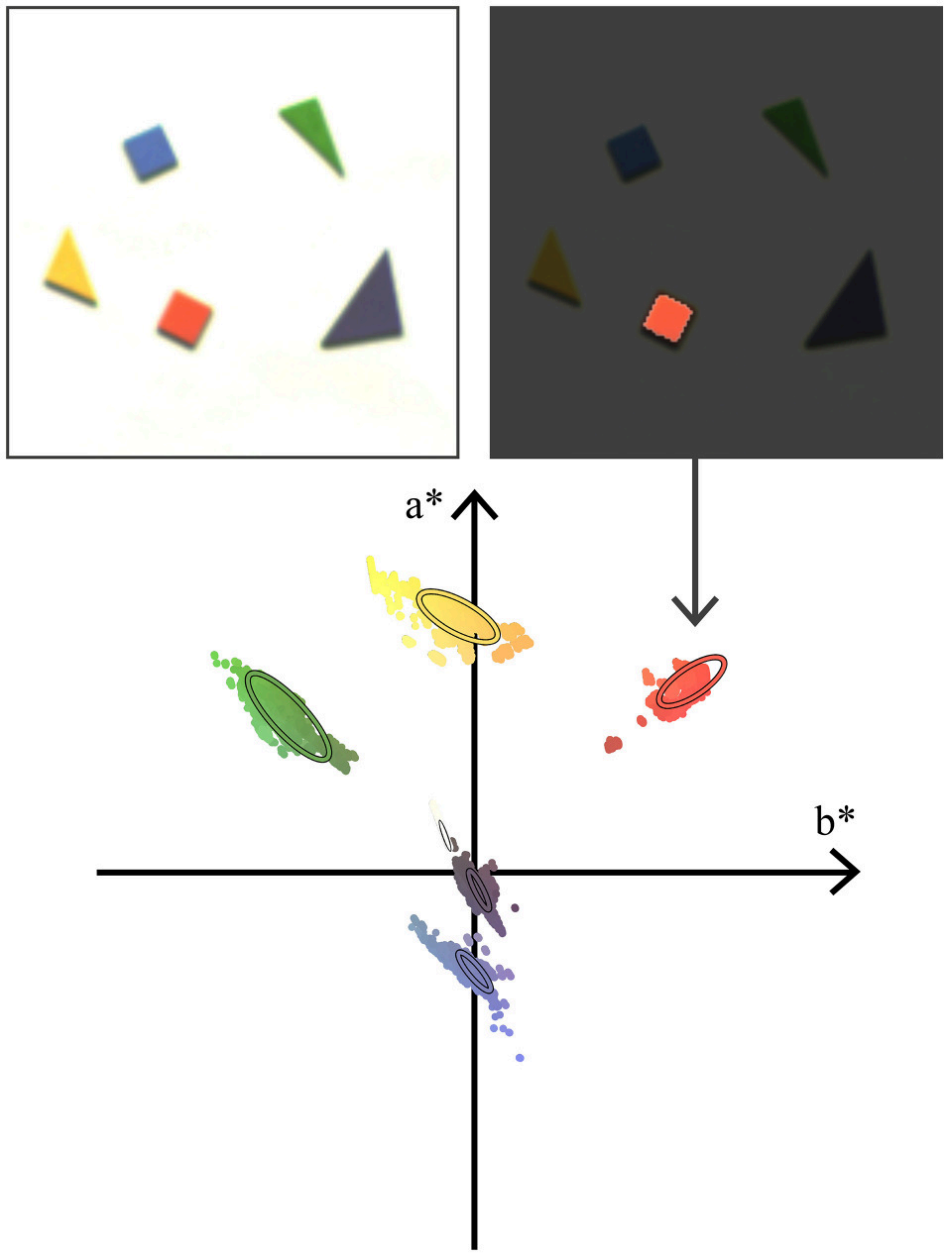
In order to build color signatures for a set of wooden pieces, we accumulate noise-generated events from a static scene. We use the resulting image to build a mask labeling a specific piece (here, the orange one). All the color events associated with the mask's pixels are converted to the CIEL*a*b* space and projected on the a*b* plane. The projected points are used to estimate a bivariate normal distribution, which we call the signature. The bottom diagram shows 95% confidence ellipses of the wooden pieces' signatures in the a*b* plane.

The experiment presented Figure 6 illustrates the method to determine the signature of actual objects. Five colored wooden pieces placed on a white background are recorded. Even though the scene is static, the ATIS cameras' noise triggers exposure measurements which are converted to color events. We associate a pixel set—or mask—to each wooden piece. The color events generated by this pixel set make up the *S* sequence used to fit a signature. The color signature for the background is evaluated as well.

### 2.5. Tracking

After determining the five wooden pieces' signatures, we consider a scene with the same pieces moving. Let *X* be the continuous bivariate random variable which associates each color event with its a^*^ and b^*^ components:

(6) $$\begin{array}{l} X:\left(\mathbb{N},{\mathbb{R}}^{+},{\mathbb{R}}^{3}\right)\to {\mathbb{R}}^{2}\\ \;e_{i,t}\mapsto \left(\begin{array}{c} a\\ b \end{array}\right) \end{array}$$

We note $x=\left(\begin{array}{c} a\\ b \end{array}\right)$ a color event's a^*^ and b^*^ components.

Writing *O*_*j*_ for the probabilistic event “the color event was generated by the object *j*,” the Bayes' theorem yields the probability that the object *j* generated the considered color event:

(7) $$\begin{array}{r} P\left(O_{j}|\text{X}=\text{x}\right)=\frac{f_{O_{j}}\left(\text{x}\right)P\left(O_{j}\right)}{\overset{m-1}{\underset{j\;=\;0}{\sum }}f_{O_{k}}\left(\text{x}\right)P\left(O_{k}\right)} \end{array}$$

*f*_*O*_*j*__ is the probability density function of the bivariate normal distribution associated with the object *j*, and *m* is the number of objects.

The color event is associated with the object with the largest probability to be the source of the event. Assuming an identical probability $P\left(O_{j}\right)=\frac{1}{m}$ for each object to generate an event (six in the wooden pieces example, background included), we simply need to find the index *j* maximizing *f*_*O*_*j*__(**x**), given by:

(8) $$\begin{array}{r} f_{O_{j}}\left(\text{x}\right)=\frac{1}{2\pi \left|\Sigma_{j}\right|}e^{-\frac{1}{2}{\left(\text{x}-\mu_{j}\right)}^{T}\Sigma_{j}^{-1}\left(\text{x}-\mu_{j}\right)} \end{array}$$

where **μ**_*j*_ and **Σ**_*j*_ are the object *j*' signature' mean and covariance matrix.

In order to track the objects, we use a moving mean algorithm. Each object is given a center $p_{j}=\left(\begin{array}{c} x_{j}\\ y_{j} \end{array}\right)$, where *x*_*j*_ and *y*_*j*_ are the object's mean coordinates in the screen referential. When an event *e*_*i,t*_ is generated, the mean associated with the object minimizing expression 8 is updated. The new mean ${\text{p}}_{j}^{\prime }$ is calculated from the previous mean and the event as:

(9) $$p_{j}^{\prime }=\lambda p_{j}+\left(1-\lambda \right)x_{i}$$

where **x_i_** is the coordinates in the screen referential of the pixel which generated the event. λ is an inertia parameter ranging from zero to one. λ is generally given a value close to (1−10^−3^). The larger λ, the more robust to noise the tracking. However, large λ values yield more latency and deteriorate the algorithm's ability to account for small variations.

We take into account the camera noise with a spatio-temporal activity filter. Once an event is associated with an object, we count the number of prior events associated with the same object that were generated less than one second before in a six-by-six square window around the event's position. Only events with at least thirty neighbors in this spatio-temporal window are taken into account for updating the object's mean position. Increasing the required count decreases the number of false positive events, while increasing the number of false negative events.

## 3. Results

**Figure 7:**
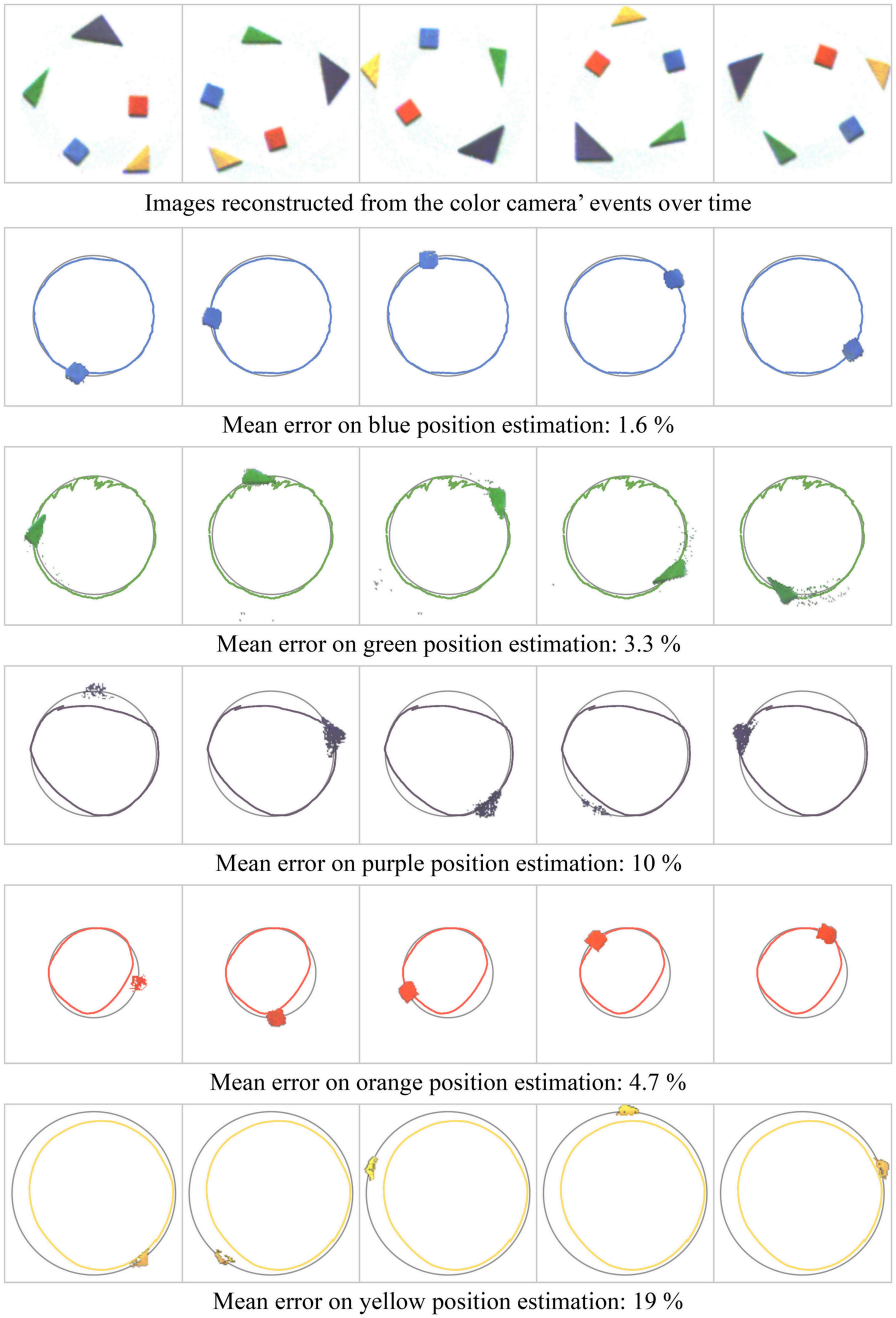
Tracking of five wooden pieces in rotation motion. Figures show the pieces motions estimated with our method (colored trajectories) and the ground truth (gray trajectories) for a whole rotation. The mean error for each object is the average distance between the estimated object's mean position and the ground truth as a ratio of the yellow object's trajectory's radius. The observed errors derive from the compromise between noise robustness and accuracy imposed by the moving mean algorithm.

We applied the color tracking algorithm to three experiments. Videos illustrating the associated results are provided as Supplementary Materials. The first experiment is recorded under laboratory-controlled conditions. Five colored wooden pieces are placed on a rotating surface captured with a static sensor. Figure 7 shows the results compared to the ground truth, evaluated with a contour tracing algorithm (Suzuki and be, 1985). The objects and their centers are identified on images reconstructed from the color camera's events. For each event, we calculate the distance between the associated object's estimated mean and its ground truth. The mean distance is given for each object as a fraction of the yellow object's trajectory's radius. We are able to estimate the objects trajectories using only color data. The moving mean algorithm's λ parameter is identical for all the objects, empirically set to (1−10^−3^). A compromise must be reached between noise robustness and accuracy. Reducing the parameter's value would improve results for the purple object while degrading the results for the green one.

**Figure 8:**
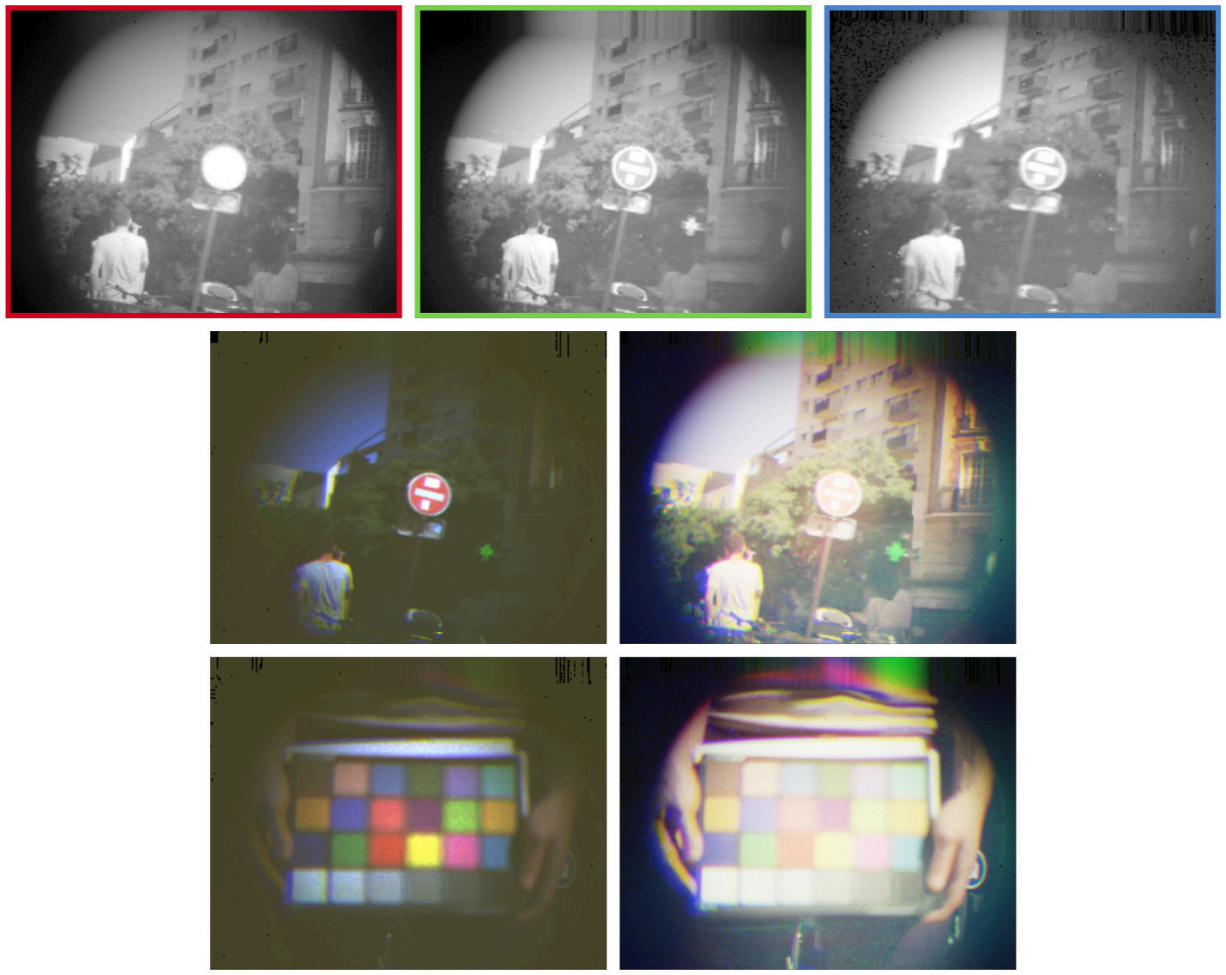
The figure's top row shows reconstructed frames from events acquired by each sensor of the three-chip event-based camera. The gray levels are tone-mapped with a logarithmic function in order to be displayed on a regular screen. The bottom-left frames are reconstructed with the linear color model presented in the methodology. The colors are properly reconstructed, but very little detail is left in dark areas. The bottom-right frames use the channels' logarithmic tone-mappings as their color channel, yielding incorrect colors but much more details in dark areas.

The second experiment consists of a moving camera in an urban scene containing a red road sign and a green pharmacy neon light. The corresponding color signatures are learnt from an initialization step. The latter uses data from a one-second sequence taking place before the experiment's recording. Figure 8 illustrates the associated color reconstruction process. Due to the scene's high dynamic range, the linear color model yields little detail in the dark areas of reconstructed frames. Therefore, we generate color frames for display purposes by applying a logarithmic tone-mapping on each channel independently. This operation yields incorrect colors, but shows much more detail in dark areas. The tracking algorithm is not influenced by this operation since it uses colors calculated by the linear model, as presented in our methodology.

**Figure 9:**
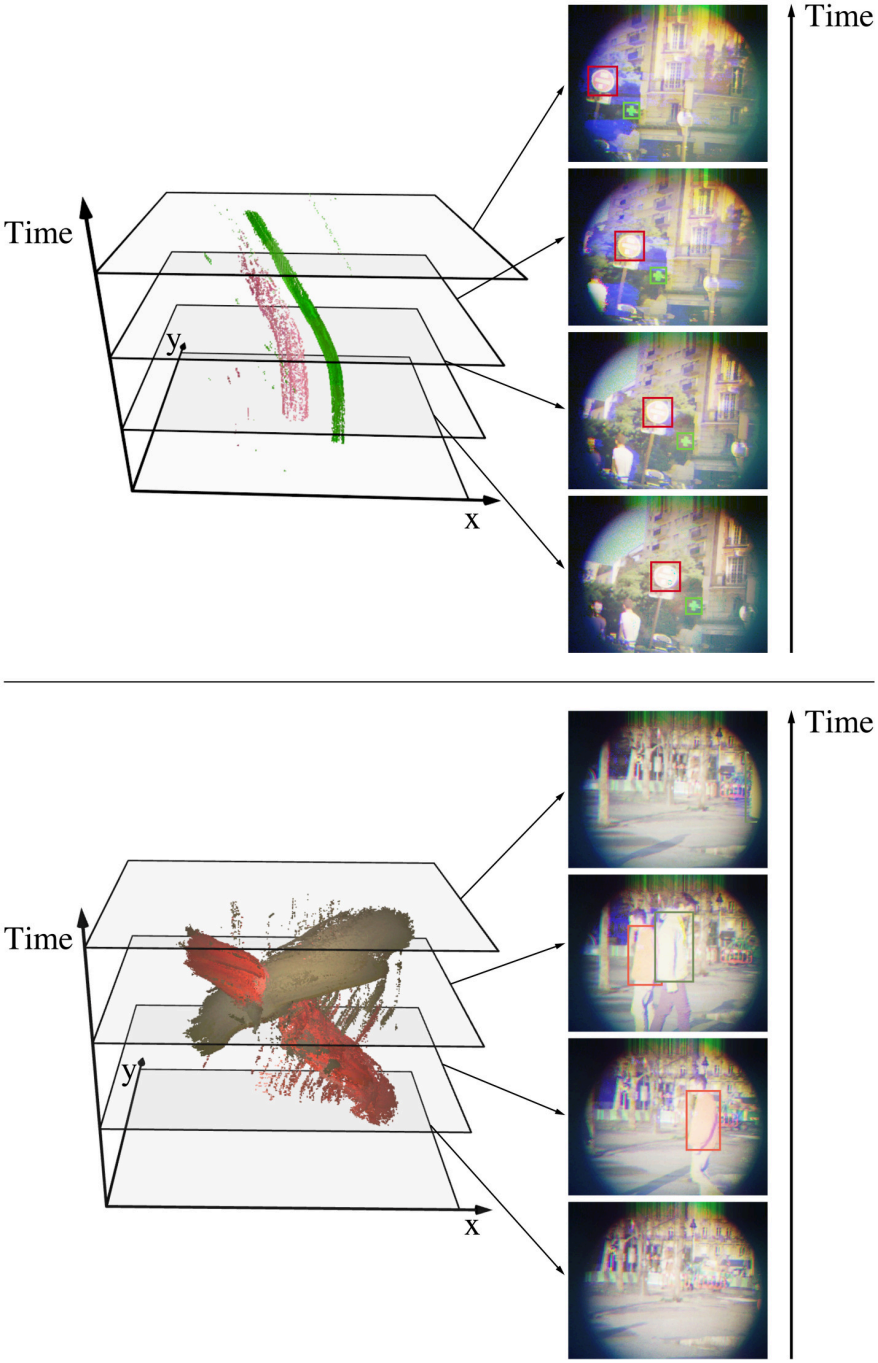
We consider two outdoors scenes to assess the tracking algorithm performance: a moving camera acquiring a red road sign and a green pharmacy sign **(Top)**, and a static camera recording pedestrians wearing colored sweaters **(Bottom)**. The color signatures for the objects are calculated from similar scenes. The point clouds show color events where *f*_*O*_ is larger than 10^−5^ for one of the objects. These events are used to update the estimated center of the associated object with a moving mean algorithm where λ = 1−10^−3^. The tracked object are framed on the reconstructed frames for better visualization.

**Figure 10:**
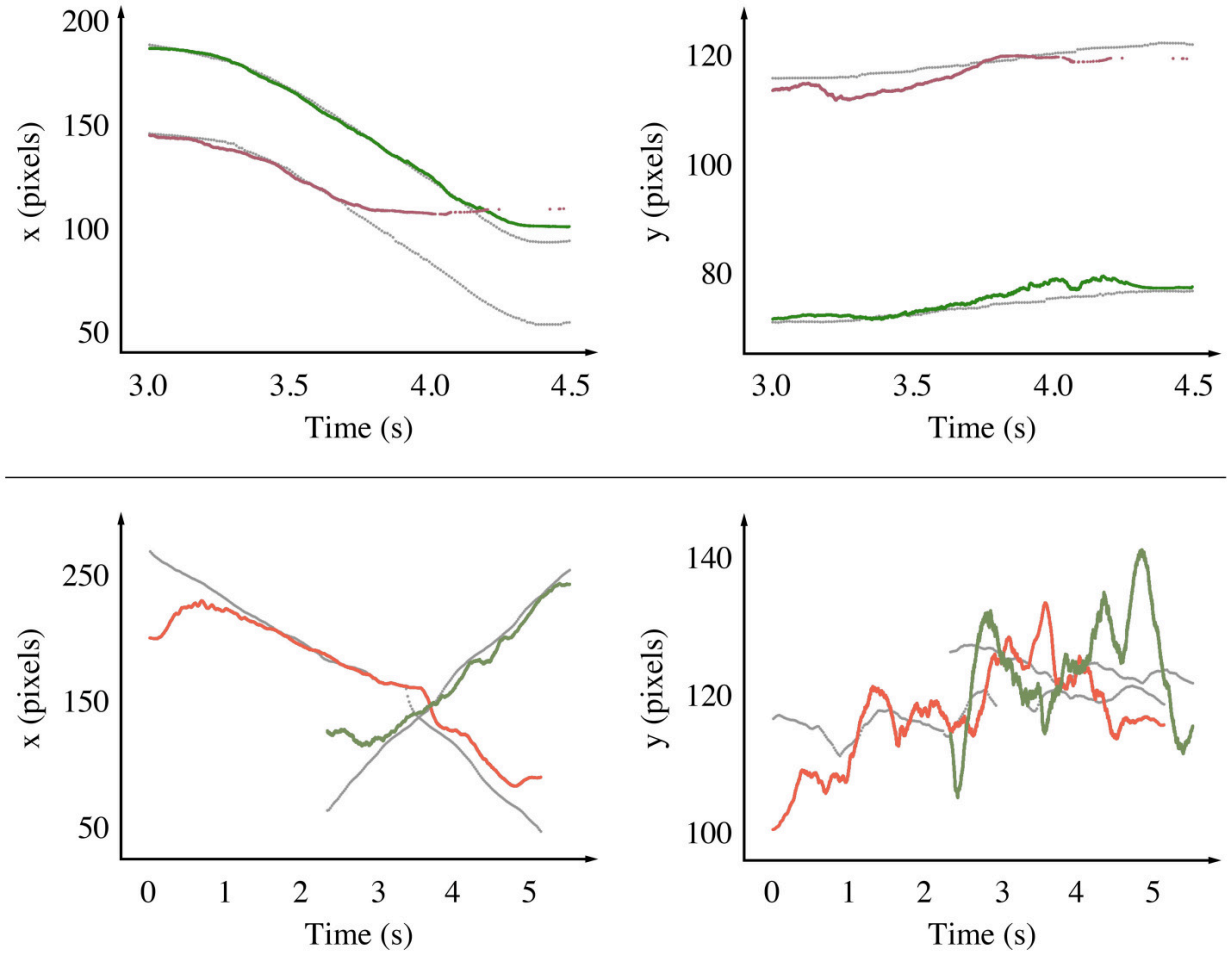
We compare the estimated position of the tracked objects with the ground truth along the x and y axis as functions of time for the road signs experiment **(Top)** and the pedestrians experiment **(Bottom)**. The events' timestamps are corrected to account for the delay induced by the mean-shift algorithm, effectively comparing dynamics rather than absolute values. Tracking is degraded near the edges, especially for the red stop sign (**Top**, x axis, after 3.6 s), which is a consequence of our prototype's optical aberrations. Since the pedestrians move along the x axis, motion along the y axis is relatively small, making the noise appear stronger.

The third experiment takes place in an urban scene as well. It consists of two pedestrian wearing colored sweaters walking in front of the sensor. Figure 9 shows the segmented events for both urban experiments, while Figure 10 compares the estimated trajectories with the ground truth. The latter is computed with the contour tracing algorithm provided by Suzuki and be (1985) as well. We remind the reader that this technique exploits shape rather than color: spatial constrains yield more robust results, but require a more complex algorithm. The estimated mean position lags behind the ground truth, which is a consequence of the moving mean algorithm. In order to assess the dynamics of our results, we compensate the lag for the road signs experiment and shift the position's reference for the pedestrians one. Table 1 summarizes the mean errors and standard deviations along the *x* and *y* axis for both urban scenes. We observe degraded performances for objects near the sensor's edges, which can be attributed to sensor limits. On the one hand, the ATIS camera used in the assembly lacks sensitivity to blue wavelengths, which is reflected by longer integration times for this component. This leads to timestamp differences between channels, which result in incorrect color reconstructions. On the other hand, our prototype three-chip color camera exhibits optical aberrations which degrade the signal near the edges.

**Table 1:** The mean error between the estimated position and the ground truth is evaluated for each tracked object in the outdoor scenes.

**Object**	**Mean error (pixels)**	
	**x**	**y**
Green sign	2.18	1.30
Red sign	18.4	2.09
Orange sweater	14.5	4.58
Brown sweater	11.9	6.20

*The larger errors along the x axis for three out of four object can be attributed to the optical aberration near the sensors' edges*.

## 4. Discussion

The event-based three-chip color camera is the first working prototype of an event-based sensor able to acquire absolute color information: the sensor generates packets of data carrying the luminance value integrated over a small time interval. By contrast, the event-based color pixel designed 6 years ago (Berner and Delbruck, 2011) and the DVS camera with a Bayer matrix built in early 2017 (Moeys et al., 2017) can only detect color variations: they send the same message regardless the variation magnitude, and require heavy calculations to retrieve the absolute luminance. Even though our prototype is still at an early stage, we manage to track colored objects in several scenes, using only the color information generated by the sensor. Thanks to the capture of absolute luminance, very little computational power is required to label the events. Therefore, the algorithm is a good candidate for the first stage of a complex chain of computations achieving a higher level task. It proves that color information alone is enough to achieve tracking with event-based cameras. The advantages of the event-based color sensor presented in this work over frame-based color cameras are similar to the advantages of gray event-based sensors over gray frame-based sensors: carrying out part of the computation on the sensor yields a natural data compression, with an increased temporal resolution. Both greatly reduce the required amount of computation for the processor. However, a proper use of the generated data requires a re-design of most computer vision algorithms.

The presented prototype can find applications in embedded systems. When low latency and low power consumption are required—as an example, with drones—conventional vision sensors show limits which can be overcome with event-based cameras. Fast color segmentation on a drone can be useful for several tasks, such as target detection and tracking or environment mapping. Moreover, the high dynamic range of the ATIS camera tackles the luminance adaptation issue, particularly troublesome for self-driving cars. Color makes road sign segmentation and recognition much easier on such systems.

The use of spatial information is out of the scope of this work. However, it should allow for a more robust algorithm thanks to data fusion, and is considered as this work continuation. We also identify several areas of improvement for the sensor that would benefit the algorithm's results. These improvements require hardware development. On the one hand, one of the event-based three-chip color sensor's weaknesses is its lack of sensitivity to short wavelengths. This limitation is shared by most silicon-based photo-detectors, but is aggravated by the ATIS's low sensitivity to low light. Therefore, better results would be achieved with increased sensitivity. On the other hand, the three-chip event-based prototype exhibits optical limits, such as color aberrations near the edges and vignetting. These shortcomings are caused by the large size of the electronics boards used by the ATIS sensors to communicate with the computer: the sensors are 18 × 18 mm, while the board are 50 × 50 mm. They impose large distances between the light entry and the sensors, which in turn require the objectives to be placed after the beam splitters. The small defects in the beam splitters' angles and positioning are responsible for the visible aberrations. The circular field of view is a consequence of a compromise reach with the off-the-shelf components used in the assembly: the input hot mirror results in a partially masked field of view, however a larger one would increase the amount of reflections inside the sensor's casing, which degrades capture. Designing a dedicated electronics board matching the sensor size would allow a casing reduction large enough to place as single objective before the light entry, hence alleviating the optical aberrations. Another solution consists in using a single array of pixels with a Bayer matrix. However, the latter requires designing a chip from the ground up, as Bayer matrix placing is part of the pixel building process.

Assuming the design of a new chip, it would be interesting to consider the following problem. Both the sensor presented in this work and the existing event-based color sensors digitize the analog light signal into events for each channel independently. Processing is then performed on the generated events, including color merging. The parallel drawn with the human eye for such sensors (Posch et al., 2011) ignores part of the eye complexity, including data passed between pixels through the horizontal cells. This data appears to be analog rather than digital. Implementing such a data transfer in the next generation of color neuromorphic vision sensors may be the key to acquiring color information efficiently. It may also help overcoming the following paradox in computer vision: for segmenting natural scenes, color, though helpful, provides a generally small advantage (Hansen and Gegenfurtner, 2017). However, it requires dealing with three times as much data. Assuming an access to the extra power required to deal with this data, one is generally better off with a more complex algorithm working on gray levels. Merging colors on the analog level may help reducing the amount of generated data without tainting its quality.

## Author contributions

AM, S-HI, and RB contributed conception and design of the study. AM devised the theoretical model, with substantial contributions from CS-C. AM and CS-C carried out the experiments. AM analyzed the results, and wrote the first draft of the manuscript. All authors contributed to manuscript revision, read and approved the submitted version.

### Conflict of interest statement

The authors declare that the research was conducted in the absence of any commercial or financial relationships that could be construed as a potential conflict of interest.

## Supplementary material

Supplementary Video 1 Simple shapes.

Supplementary Video 2 Road signs.

Supplementary Video 3 Pedestrians.
